# Functional Mitral Regurgitation in the Transcatheter Era: Diagnostic and Therapeutic Pathways

**DOI:** 10.3390/jpm15080372

**Published:** 2025-08-13

**Authors:** Francesca Maria Di Muro, Luigi Spadafora, Angela Buonpane, Francesco Leuzzi, Giulia Nardi, Eduardo Bossone, Giuseppe Biondi Zoccai, Tiziana Attisano, Francesco Meucci, Carlo Di Mario, Carmine Vecchione, Gennaro Galasso

**Affiliations:** 1 Cardiology Unit, Cardiovascular and Thoracic Department, University Hospital “San Giovanni di Dio e Ruggi d’Aragona”, Largo Città di Ippocrate, 84131 Salerno, Italyggalasso@unisa.it (G.G.); 2 Department of Structural Interventional Cardiology, Careggi University Hospital, 50134 Florence, Italy; 3 Department of Medical-Surgical Sciences and Biotechnologys, Sapienza University of Rome, 00161 Latina, Italy; spadafora.1697031@studenti.uniroma1.it (L.S.); giuseppe.biondizoccai@uniroma1.it (G.B.Z.); 4 Ospedale Isola Tiberina-Gemelli Isola, 00186 Rome, Italy; 5 Department of Public Health, University of Naples “Federico II”, Via Pansini, 5, 80131 Naples, Italy; 6 Maria Cecilia Hospital, GVM Care & Research, 48033 Cotignola, Italy

**Keywords:** functional mitral regurgitation, multimodal imaging, percutaneous treatment

## Abstract

Functional mitral regurgitation (FMR) is a common condition with significant prognostic implications, primarily driven by left atrial or ventricular remodeling secondary to ischemic or non-ischemic cardiomyopathies. While guideline-directed medical therapy (GDMT) remains the cornerstone of management, reducing mitral regurgitation severity in up to 40–45% of cases, additional interventions are often necessary. In patients where atrial fibrillation (AF) or ventricular dyssynchrony due to abnormal electrical conduction contributes to disease progression, guideline-directed AF management or cardiac resynchronization therapy plays a pivotal role. For those with persistent moderate to severe MR and unresolved symptoms despite optimal GDMT, percutaneous intervention may be warranted, provided specific clinical and echocardiographic criteria are met. This review highlights a precision-medicine approach to patient selection for transcatheter treatment of functional mitral regurgitation (FMR), emphasizing the integration of clinical characteristics with advanced multimodal imaging, including echocardiography, cardiac magnetic resonance, and computed tomography. In anatomically or clinically complex cases, complementary use of these imaging modalities is essential to ensure accurate phenotyping and procedural planning. Once a suitable candidate for percutaneous intervention has been identified, we provide a detailed overview of current transcatheter strategies, with a focus on device selection tailored to anatomical and pathophysiological features. Finally, we discuss emerging technologies and evolving therapeutic paradigms that are shaping the future of individualized FMR management.

## 1. Introduction

Functional mitral regurgitation (FMR) represents a complex and heterogeneous clinical entity, arising not from intrinsic valvular pathology but as a consequence of adverse left atrial or ventricular remodeling, most commonly due to ischemic or non-ischemic cardiomyopathy [1,2,3]. Affecting over four million individuals in the United States alone, and nearly 10% of those aged over 75, FMR is independently associated with excess morbidity, heart failure hospitalizations, and a mortality rate exceeding 50% at five years [4,5,6]. Despite advancements in pharmacologic and device-based heart failure therapies, a substantial proportion of patients with moderate-to-severe FMR remain symptomatic and at high risk for adverse outcomes [7,8].

In recent years, the field of cardiovascular medicine witnessed a paradigm shift toward precision-based approaches across a wide spectrum of interventions, including structural, coronary, and rhythm management [9]. Advances in multimodal imaging, patient-specific computational modeling, and biomarker-guided risk stratification have enabled more tailored treatment strategies, improving outcomes in both valvular and non-valvular heart disease [10,11,12]. Recent developments in transcatheter mitral valve interventions—particularly transcatheter edge-to-edge repair (TEER)—have fundamentally redefined therapeutic options for patients with FMR deemed high-risk or ineligible for surgery [13,14,15,16]. However, this progress has also exposed the inadequacy of a *one-size-fits-all* strategy. The mechanistic diversity of FMR, now broadly categorized into atrial, ventricular, and mixed phenotypes, necessitates a paradigm shift toward personalized, anatomy- and physiology-guided treatment selection [17]. The integration of advanced multimodal imaging—including echocardiography, cardiac magnetic resonance imaging, and cardiac CT—is indispensable for accurate phenotyping and procedural planning [18,19,20].

In this review, we critically examine the current landscape of percutaneous therapies for FMR, emphasizing individualized patient selection informed by clinical, anatomical, and pathophysiological characteristics. We explore the evidence supporting device-specific strategies, summarize procedural steps, and highlight emerging technologies poised to expand the therapeutic frontier.

## 2. Identifying Patients Likely to Benefit from Mitral Percutaneous Intervention

FMR is a frequent and clinically significant sequela of heart failure (HF) with reduced or mildly reduced ejection fraction. Unlike primary mitral disease, FMR arises from atrial or ventricular remodeling that distorts the mitral apparatus, leading to impaired leaflet coaptation in the absence of intrinsic valvular pathology [21]. The development of moderate-to-severe or severe FMR in patients with HF is strongly associated with exacerbated symptoms, diminished exercise tolerance, higher rates of hospitalization, and adverse long-term outcomes [22,23,24].

**Figure 1:**
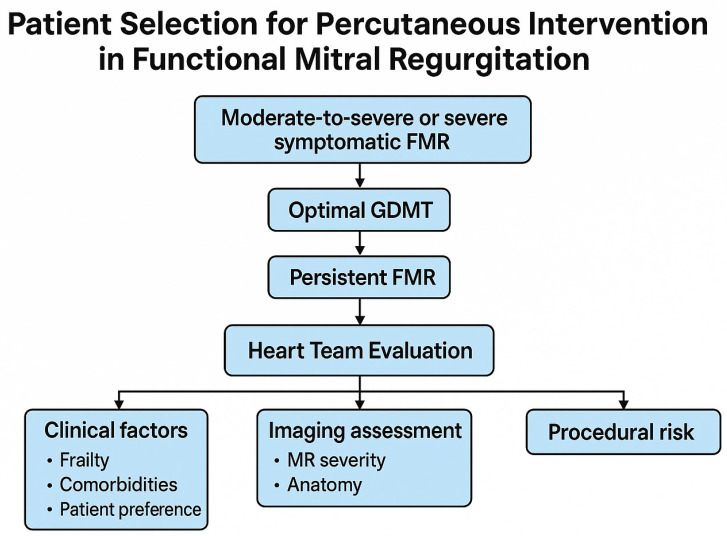
Patient selection for transcatheter treatment of functional mitral regurgitation. Abbreviations: FMR = functional mitral regurgitation; GDMT = guideline-directed medical therapy; MR = mitral regurgitation.

According to the 2021 ESC Guidelines on Heart Failure and the 2021 ESC/EACTS Guidelines for the Management of Valvular Heart Disease, the cornerstone of FMR management remains the optimization of guideline-directed medical therapy (GDMT) [25,26]. This includes the full implementation of therapies with proven prognostic benefit—such as beta-blockers, renin–angiotensin system inhibitors (including ARNIs), mineralocorticoid receptor antagonists, and SGLT2 inhibitors—as well as device-based treatments like cardiac resynchronization therapy (CRT), when indicated. The effect of these therapies on left ventricular remodeling and mitral regurgitation severity must be carefully reassessed after their introduction, as many patients experience significant improvement in MR severity with optimized medical care alone (Figure 1).

Spinka et al. evaluated the impact of GDMT titration on FMR severity in a cohort of 261 patients with HF. They reported that 39.3% of patients achieved at least a one-grade reduction in FMR severity, which correlated with evidence of reverse cardiac remodeling and clinical improvement. Furthermore, in multivariate analysis, GDMT titration was independently associated with FMR improvement (adjusted OR 2.91, *p* = 0.007), with the most pronounced effects observed in those receiving angiotensin receptor–neprilysin inhibitors (ARNi) or combination regimens incorporating renin–angiotensin system inhibitors (RASi), beta-blockers, and mineralocorticoid receptor antagonists (MRAs) [27].

Similarly, in the EFFORT trial, treatment with ertugliflozin significantly reduced FMR severity in patients with HF and mildly to moderately reduced left ventricular ejection fraction (LVEF 35–50%), accompanied by improvements in regurgitant volume, left atrial volume, and left ventricular global longitudinal strain (GLS) over a 12-month period [28]. In a preclinical model of MR-induced HF, dapagliflozin demonstrated beneficial effects by improving LV function, reducing atrial fibrillation susceptibility, and attenuating both myocardial fibrosis and endoplasmic reticulum stress [29]. Once patients remain symptomatic despite optimal and sustained GDMT, transcatheter mitral valve interventions may be warranted.

### 2.1. Multidisciplinary Evaluation and the Heart Team Approach

Timely referral to a multidisciplinary Heart Team (HT) is essential for evaluating patients with persistent FMR not responsive to GDMT. As emphasized by ESC/EACTS guidelines, individualized, patient-centered decision-making should transcend anatomical feasibility to incorporate clinical status, comorbidity burden, frailty, and patient goals of care (Table 1) [25,26]. The HT should include heart failure specialists, interventional cardiologists, cardiac surgeons, imaging experts, and anesthetists, with geriatricians or palliative care specialists involved when frailty or limited life expectancy is a concern [30,31].

The value of this multidisciplinary approach has been demonstrated in clinical cohorts [32]. Külling et al. evaluated 400 patients with significant mitral regurgitation who underwent Heart Team evaluation. Based on clinical risk profile and anatomical suitability, patients were allocated to transcatheter edge-to-edge repair (TEER) with the MitraClip device (45%), surgical repair (46%), or surgical replacement (9%). Despite higher baseline risk, MitraClip patients experienced low in-hospital mortality (3.4%), while surgical repair was associated with the best 4-year survival and lowest adverse event rates. These findings highlight the HT’s ability to match treatment strategy with patient characteristics, thereby optimizing outcomes [33].

A critical element in this evaluation is the assessment of frailty, which independently affects prognosis regardless of procedural success [34]. In a study by Metze et al., among 213 patients undergoing percutaneous mitral valve repair, 45.5% were classified as frail. While procedural success and symptomatic improvement were similar across frailty groups, frail patients experienced significantly higher 6-week mortality (8.3% vs. 1.7%) and worse long-term outcomes, despite reporting better quality-of-life gains [35]. Similarly, Rios et al. analyzed over 10,000 patients undergoing TEER with MitraClip in the U.S. National Inpatient Sample and found that frailty—present in 10.6%—was independently associated with increased in-hospital mortality (OR 3.70), respiratory complications, sepsis, transfusion needs, longer hospitalizations, and higher healthcare costs [36]. These data reinforce the importance of frailty as a prognostic and economic modifier and the need to incorporate it into HT deliberations [37].

Ultimately, the Heart Team not only enables optimal procedural planning but also ensures that treatment decisions align with the patient’s values, preferences, and clinical trajectory—principles at the heart of precision medicine and contemporary management of FMR. Furthermore, nuanced discussions within the Heart Team help avoid therapeutic futility by recognizing patients in whom intervention is unlikely to improve outcomes.

### 2.2. The Role of Multimodal Imaging in Patient Selection

Accurate and comprehensive imaging assessment is pivotal for the appropriate selection of patients with FMR who may benefit from percutaneous intervention [38]. Multimodality imaging plays a dual role: first, to determine the severity and mechanism of mitral regurgitation; second, to assess anatomical feasibility and procedural suitability for transcatheter therapies, particularly TEER and, in selected cases, transcatheter mitral valve replacement (TMVR) [38,39].

Transthoracic echocardiography (TTE) remains the first-line imaging modality, offering crucial insights into both MR severity and its underlying mechanism. Key parameters include vena contracta width, effective regurgitant orifice area (EROA), regurgitant volume, and regurgitant fraction. These quantitative indices, although subject to technical limitations, are essential to establish the diagnosis of moderate-to-severe or severe MR according to guideline definitions. Importantly, echocardiographic assessment must be contextualized within the framework of LV volumes and function, as loading conditions can dynamically influence MR severity [40]. An MR jet that appears “severe” in isolation may be proportionate to advanced LV remodeling and may not be an appropriate target for intervention [41].

To better characterize valvular anatomy and improve procedural planning, transesophageal echocardiography (TEE)—especially with three-dimensional (3D) imaging—is essential. TEE enables high-resolution visualization of leaflet morphology, coaptation geometry, and sub-valvular apparatus [42,43]. A comprehensive overview of the TEE views used during FMR assessment is provided in Table 2. For TEER, specific anatomical features are predictive of procedural success and durability: a coaptation length of at least 2 mm and a coaptation depth less than 11 mm are considered favorable, though newer-generation devices with extended grasping capabilities (e.g., MitraClip XTR, G4, and PASCAL) have broadened the anatomical window [44]. The presence of severe leaflet tethering, mitral annular calcification, and eccentric or multi-jet MR may pose technical challenges and may influence device choice or even preclude TEER altogether [45].

In certain anatomies, additional considerations are necessary [44]. For example, the presence of leaflet clefts or fissures—either congenital or acquired—can mimic central jets but may prevent adequate leaflet coaptation after TEER, particularly if not recognized on preprocedural 3D TEE [45]. These subtle defects may lead to residual MR despite technically successful device deployment. Similarly, multi-jet MR, especially when involving eccentric or posteriorly directed jets, requires careful mapping to ensure that all regurgitant orifices can be addressed by a feasible clipping strategy; failure to identify secondary jets may result in under-treatment [43]. Other anatomical variants that may challenge TEER include mitral annular disjunction, which affects device anchoring and the stability of leaflet approximation, and focal mitral calcification, particularly when located at leaflet grasping zones, which can hinder clip closure or increase the risk of leaflet injury [46,47]. Moreover, marginal or asymmetric leaflet prolapse, though more typical of primary MR, can coexist with ventricular remodeling in mixed etiologies and requires differentiation, as these patients may be more appropriate for surgical intervention or alternative percutaneous strategies [48].

Cardiac magnetic resonance (CMR) serves as a valuable adjunct in selected patients, especially when echocardiographic windows are suboptimal or in case of quantification discrepancies [49]. CMR provides gold-standard measurements of LV volumes, ejection fraction, and regurgitant volume and fraction, without relying on geometric assumptions. This becomes particularly useful in patients with eccentric or multiple MR jets, where echocardiography may underestimate regurgitation severity. Moreover, CMR allows for tissue characterization through late gadolinium enhancement, which identifies myocardial fibrosis [50]. The extent and distribution of fibrosis may influence both procedural success and long-term outcomes, as patients with extensive non-viable myocardium may derive limited benefit from MR correction [51]. CMR is also increasingly used in clinical research to refine phenotyping of patients with FMR and HF, and may help distinguish between patients with truly disproportionate MR versus those in whom MR is merely a marker of advanced LV remodeling [52].

Cardiac computed tomography (CT), while traditionally less central in the evaluation of secondary MR, has gained importance in the era of TMVR. Multidetector CT provides high-resolution anatomical reconstructions that are essential for accurate measurement of the mitral annulus, assessment of annular and leaflet calcification, and evaluation of spatial relationships between the mitral valve, left ventricular outflow tract (LVOT), and surrounding structures. One of the key steps in pre-TMVR planning is the prediction of the post-procedural neo-LVOT area, which, if critically narrowed, may result in life-threatening obstruction [53,54,55]. CT imaging is also used to predict device–annulus fit, assess risk of paravalvular leak, and determine access routes. In the future, the integration of CT-derived computational modeling, including virtual device deployment and fluid dynamics simulation, may further refine patient selection and procedural strategy.

The integration of these imaging modalities within a multidisciplinary approach allows for comprehensive phenotyping and personalized treatment planning. A patient with severe FMR, preserved leaflet anatomy, and viable myocardium but modest LV dilatation may be an ideal candidate for m-TEER. Conversely, a patient with extreme leaflet tethering, extensive annular dilation, and transmural myocardial fibrosis may be better suited for alternative therapies or may represent a scenario of procedural futility.

Finally, beyond valvular and ventricular imaging, both coronary CT angiography and invasive coronary angiography (in cases of limited image quality) represent critical components of the preprocedural workup in patients undergoing TEER or TMVR. These modalities enable a comprehensive assessment of the presence, extent, and complexity of concomitant coronary artery disease (CAD), informing the need for and timing of percutaneous coronary intervention when indicated.

In patients with extensive CAD, where myocardial ischemia is believed to play a key role in the pathophysiology of MR, revascularization should be prioritized, followed by a period of stabilization (typically 1 to 3 months) and optimization of GDMT. If severe MR persists despite optimal therapy, a comprehensive, multidisciplinary reassessment is warranted to guide the selection between TEER and TMVR [25,26,56].

In contrast, in patients with CAD that does not appear to account for the severity of MR, no clear evidence currently supports routine revascularization, and therapeutic decisions should rely on individualized Heart Team evaluation. Factors such as lesion complexity, ischemic burden, and potential clinical benefit must be carefully weighed. Further evidence is needed to define the role and optimal timing of percutaneous coronary intervention in this context, particularly when MR remains severe despite optimized medical therapy.

## 3. Device Selection and Procedural Steps

Transcatheter interventions for FMR can be broadly categorized into two principal strategies: repair and replacement. Repair techniques are designed to selectively address distinct components of mitral valve dysfunction, ranging from leaflet pathology (e.g., prolapse or rupture) to annular dilation and sub-valvular abnormalities secondary to ventricular remodeling—thereby allowing for a tailored, anatomy-specific approach. In contrast, TMVR offers a comprehensive solution by addressing the entirety of the mitral apparatus in a single intervention [57].

### 3.1. Transcatheter Mitral Valve Repair

#### 3.1.1. Device Types

Currently, two systems are approved in the UK and Europe for M-TEER: the MitraClip system (Abbott) and the PASCAL system (Edwards Lifesciences), each engineered with distinct design features to accommodate a wide spectrum of anatomical and pathological scenarios.

The MitraClip system, now in its fourth generation, includes four device variants (NT, XT, NTW, XTW), offering combinations of arm lengths (9 or 12 mm) and widths (4 or 6 mm). These cobalt–chromium arms are paired with nitinol-based grippers featuring frictional elements for enhanced leaflet engagement. The system supports independent gripper actuation (controlled gripper actuation, CGA) and continuous left atrial pressure monitoring, enabling refined procedural control. Extended-arm devices (XT/XTW) are well-suited for large coaptation gaps or flail segments but may increase leaflet tension, particularly in calcified or restricted anatomies—although registry data have not shown higher complication rates [58,59,60].

The PASCAL platform, introduced in 2016, incorporates two curved nitinol paddles, a central spacer to offload leaflet stress, and independently controlled clasps with integrated retention elements. The original PASCAL P10 and narrower PASCAL Ace implants offer grasping flexibility, particularly advantageous in small valves or complex anatomies requiring multiple devices. The recently released PASCAL Precision system includes modifications aimed at enhancing catheter steerability and procedural stability [61,62]. Together, Mitra-Clip and PASCAL provide complementary tools for individualized mitral repair, with device selection guided by leaflet morphology, valve area, coaptation geometry, and regurgitant jet characteristics [63].

#### 3.1.2. Evidence from Clinical Trials

So far, the role of M-TEER with the Mitraclip system in patients with FMR has been explored in three major randomized controlled trials—MITRA-FR, COAPT, and RESHAPE-HF2—each contributing uniquely to our understanding of which patients derive the greatest benefit from this intervention [16].

The MITRA-FR trial enrolled 304 patients with symptomatic HF and severe secondary MR, defined by an effective regurgitant orifice area (EROA) > 20 mm^2^ or regurgitant volume > 30 mL/beat. Eligible patients had a LVEF between 15% and 40% and were randomized to receive MitraClip plus GDMT or GDMD alone. At one year, there was no significant difference in the composite endpoint of all-cause death or unplanned heart failure hospitalization (54.6% vs. 51.3%, *p* = 0.53), and no differences in mortality or hospitalization when considered individually. These neutral findings were attributed to the inclusion of patients with more advanced ventricular remodeling and relatively milder MR—characteristics consistent with “proportionate” MR, where regurgitation reflects ventricular dysfunction rather than driving disease progression. In such patients, MR correction may confer limited clinical benefit.

In contrast, the COAPT trial employed more stringent anatomical and functional criteria, enrolling 614 patients with moderate-to-severe or severe secondary MR, LVEF between 20% and 50%, and left ventricular end-diastolic volume index ≤ 96 mL/m^2^. Patients underwent meticulous optimization of GDMT before randomization. Over five years of follow-up, MitraClip was associated with significant and durable reductions in annualized rates of HF hospitalization (33.1% vs. 57.2%; HR 0.53), all-cause mortality (57.3% vs. 67.2%; HR 0.72), and the composite endpoint of death or HF hospitalization (73.6% vs. 91.5%; HR 0.53), with a low 30-day complication rate (1.4%). These results demonstrated the long-term safety and efficacy of TEER in patients with “disproportionate” MR, where regurgitation constitutes an independent and modifiable contributor to clinical deterioration [64]. Table 3 summarizes the most commonly reported procedural complications associated with the MitraClip device in the MITRA-FR and COAPT trials.

The **RESHAPE-HF2** trial further evaluated the utility of MitraClip in a broader cohort of 505 patients with moderate-to-severe or severe secondary MR. At 24 months, the MitraClip group showed significant reductions in the primary composite of recurrent HF hospitalization or cardiovascular death (37.0 vs. 58.9 events/100 patient-years; rate ratio 0.64; *p* = 0.002), and in recurrent HF hospitalizations alone (26.9 vs. 46.6 events/100 patient-years; rate ratio 0.59; *p* = 0.002). Quality of life also improved meaningfully, with a mean 10.9-point higher Kansas City Cardiomyopathy Questionnaire–Overall Summary (KCCQ-OS) score at 12 months (*p* < 0.001).

RESHAPE-HF2 adopted more inclusive echocardiographic criteria (EROA ≥ 0.2 cm^2^, regurgitant volume ≥ 30 mL), which led to the enrollment of a more heterogeneous patient population. This broader selection likely contributed to the intermediate treatment effect observed between MITRA-FR and COAPT, highlighting the pivotal role of patient selection in determining therapeutic efficacy and offering insights into real-world applicability [65].

Collectively, these trials provide important insights into the role of M-TEER in the management of FMR. While MITRA-FR failed to demonstrate a clinical benefit in a population characterized by advanced left ventricular dilatation and comparatively less severe mitral regurgitation, both COAPT and RESHAPE-HF2 support the efficacy of TEER in appropriately selected patients. These findings reinforce the notion that the success of TEER hinges on identifying individuals in whom FMR represents a principal driver of symptoms and disease progression, rather than a mere marker of advanced ventricular remodeling [66]. The emerging concept of “disproportionate” MR, though still debated, provides a useful framework for interpreting divergent trial results [67]. Patients with more severe regurgitation, less extensive ventricular dilation, and preserved right ventricular function are more likely to derive benefit from mitral intervention.

Ultimately, optimal candidate selection must rely on an integrated assessment of anatomical, functional, and clinical parameters. In a study of 355 high-risk patients undergoing MitraClip implantation, lower baseline LVEF, higher troponin T levels, and worse pre-procedural NYHA class were independently associated with increased risk of 1-year heart failure rehospitalization [68]. Another study by Boerlage-van Dijk et al. identified four key predictors of 2-year mortality in 84 high-risk patients with severe symptomatic MR: elevated baseline NT-proBNP (≥5000 μg/L), prior valve surgery, grade ≥ 3 tricuspid regurgitation, and failure to achieve significant MR reduction post-procedure. Notably, patients with two or more adverse features had markedly reduced survival (38%) compared to those with none or one (87% and 78%, respectively) [69]. Further supporting the importance of individualized risk assessment, Kitamura et al. retrospectively analyzed 575 patients undergoing MitraClip implantation for severe FMR across two high-volume centers, comparing outcomes by etiology. While overall survival did not differ significantly between ischemic (I-FMR) and non-ischemic (NI-FMR) cohorts, distinct predictors of all-cause mortality emerged. In I-FMR patients, lower tricuspid annular plane systolic excursion (TAPSE) and higher logistic EuroSCORE were independent predictors of mortality, whereas in NI-FMR patients, older age and elevated NT-proBNP levels were most prognostic [70]. These findings underscore the heterogeneity of FMR and the importance of etiology-specific risk stratification to inform therapeutic decision-making [71,72].

The PASCAL transcatheter repair system has also been evaluated across multiple prospective studies. In the CLASP study, a multicenter single-arm trial of 124 patients with symptomatic MR (68% functional), PASCAL achieved MR ≤ 2+ in 98% and MR ≤ 1+ in 86% at one year, accompanied by marked improvements in NYHA class, exercise capacity, and KCCQ scores [73]. Results were sustained at two years [74]. The CLASP IID trial randomized 180 patients with degenerative MR to PASCAL or MitraClip. At 6 months, PASCAL demonstrated non-inferiority for the composite of major adverse events, MR reduction, and quality-of-life improvement. Notably, PASCAL yielded a higher proportion of patients with MR ≤ 1+ and a lower rate of leaflet injury, suggesting potential advantages in anatomically complex cases [75]. The 1-year results, recently reported, confirmed durable MR reduction and symptomatic improvement, with safety outcomes comparable to MitraClip [76]. Future data from the ongoing CLASP IIF, which is enrolling over 400 patients with FMR treated with PASCAL versus GDMT, will further clarify the optimal role of PASCAL in the treatment landscape and reinforce the importance of individualized device selection guided by multimodal imaging, clinical context, and anatomical complexity.

While final results are pending, current registry and real-world data indicate that PASCAL achieves procedural and clinical outcomes comparable to MitraClip, including high technical success rates, significant MR reduction, and similar short- and mid-term mortality and HF hospitalization rates. [77,78,79,80]

#### 3.1.3. Procedural Steps

**Figure 2:**
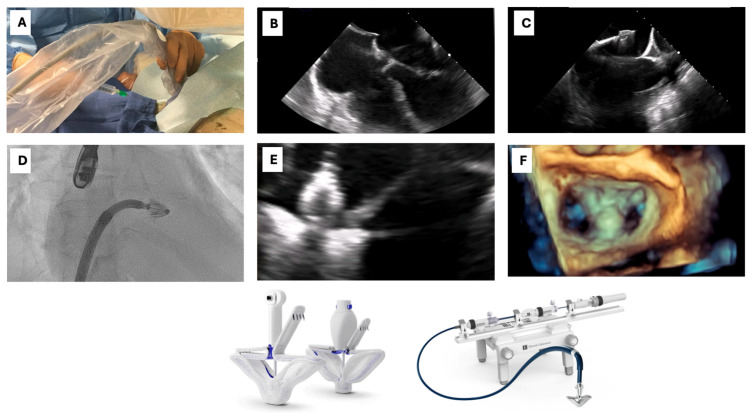
Key procedural steps of transcatheter edge-to-edge repair using the PASCAL system (Edwards Lifesciences). (**A**) Ultrasound-guided femoral venous puncture using a micropuncture set. (**B**) Tenting of the interatrial septum during transseptal puncture under transesophageal echocardiography (TEE) guidance (bicaval view), performed using a Mullins transseptal sheath and a Brockenbrough needle. (**C**) Ultrasound-guided advancement of the PASCAL delivery system (Edwards Lifesciences) into the left atrium (**D**) Fluoroscopic view showing the PASCAL implant positioned in the left atrium after successful transseptal access. (**E**) Grasping of the mitral valve leaflets using the PASCAL device under TEE guidance (mid-esophageal commissural view, ~60°), with the paddles and clasps visible. (**F**) Three-dimensional en face view from the left atrium (LA) showing the mitral valve with the PASCAL device implanted, demonstrating effective leaflet approximation and reduction of mitral regurgitation.

The m-TEER procedure can be divided into four main procedural steps (Figure 2) [81]:

(1) Vascular access
(2) Transeptal puncture
(3) Device advancement and implantation
(4) Vascular access closure

The procedure is usually performed under general anesthesia with both fluoroscopic and TEE guidance. TEE is the primary tool used to guide the procedure and clear communication of all operators with the echocardiographer being crucial to achieve procedural success.

2. Vascular access

The preferred vascular access for M-TEER is the right common femoral vein, accommodating a 24F steerable guide catheter (MitraClip) or a 22F guide sheath (PASCAL). Right-sided access is typically favored to optimize transseptal trajectory and facilitate coaxial device alignment. Following venous puncture, pre-closure with a suture-mediated device (e.g., Perclose ProGlide) is recommended to ensure reliable hemostasis post-procedure; in most cases, a single device is sufficient. In anatomically challenging cases, ultrasound-guided access and micropuncture techniques are advised to minimize vascular complications and enhance procedural safety.

2. Transeptal puncture

At this stage, the transseptal puncture system is introduced and advanced into the right atrium over a 0.035″ guidewire. The optimal puncture site is located superiorly and posteriorly along the interatrial septum, typically at a height of approximately 4.5 cm above the mitral annular plane. Precise localization is paramount, and the use of TEE in conjunction with fluoroscopy is essential to guide the operator in selecting the most appropriate puncture site based on individual patient anatomy and the underlying etiology of MR. Integrated imaging platforms that allow real-time fusion of 3D TEE and fluoroscopic images (e.g., EchoNavigator, Philips) can further enhance procedural accuracy during this critical step. The required transseptal height should be individualized: in functional MR, the target is typically located inferior to the annular plane, necessitating a more ventricular puncture; in contrast, degenerative MR due to prolapse or flail originates above the annulus, favoring a more atrial trajectory. A Brockenbrough needle is commonly employed, though in cases of septal thickening or fibrosis, electrocautery-assisted puncture may be required. Successful entry into the left atrium is confirmed by TEE and fluoroscopic imaging, and a left atrial pressure tracing is recommended both to validate positioning and to establish baseline hemodynamic parameters. In cases requiring extensive catheter manipulation or septostomy, a residual iatrogenic atrial septal defect may persist following the procedure. While typically small and hemodynamically insignificant, persistent atrial septal defects have been associated with right-to-left shunting and may warrant follow-up in selected patients. Off-label closure using standard atrial septal defect occluders has been reported in such contexts [82].

3. TEER Device Delivery and Implantation

Following successful transseptal puncture, the steerable guide catheter is advanced into the left atrium over a stiff 0.035″ guidewire positioned in the left upper pulmonary vein. The M-TEER system is continuously flushed with heparinized saline throughout the procedure to minimize the risk of air embolism. In cases of resistance to advancement due to septal stiffness or unfavorable anatomy, the use of an 18–20F dilator or balloon septostomy (10–12 mm) may facilitate device passage. Once the guide catheter is positioned 1–2 cm into the left atrium and the dilator is removed, the delivery system is introduced. Precise alignment between the markers on the steerable guide catheter and those on the delivery system is critical to maintain full control and ensure accurate device orientation. Real-time TEE and fluoroscopic guidance are employed throughout this step. Leaflet grasping and device positioning are confirmed using multiple TEE views, including the 0-degree and left ventricular outflow tract views (120–150°) to evaluate anterior–posterior alignment, the inter-commissural view (60–90°) for medial–lateral orientation, and the 3D en face view for spatial visualization of the mitral valve and confirmation of adequate leaflet insertion. While both systems enable independent leaflet grasping, subtle procedural nuances and device-specific articulation mechanisms must be considered during implantation. Following deployment of the first device, residual mitral regurgitation is assessed to determine the need for additional implants. Placement of a second device may enhance MR reduction, stabilize the initial device, and optimize hemodynamic outcomes, always balancing the risk of mitral stenosis with the goal of procedural efficacy.

4. Access closure

After removing the delivery system, hemostasis of the venous access site is typically achieved using pre-positioned suture-mediated closure devices and/or a subcutaneous “figure-of-eight” stitch. Administration of protamine may be considered to reverse anticoagulation and facilitate effective vascular closure.

Post M-TEER antithrombotic management should be tailored to the individual clinical context. In patients with a pre-existing indication for oral anticoagulation, such as atrial fibrillation or other thromboembolic conditions, anticoagulation should be resumed promptly. In the absence of such indications, single antiplatelet therapy, most commonly aspirin 100 mg daily, is generally sufficient to mitigate thrombotic risk following device implantation [83,84,85]. Antibiotic prophylaxis is advised in all patients undergoing procedures with a potential risk of transient bacteremia.

### 3.2. Transcatheter Mitral Valve Replacement

#### 3.2.1. Current Landscape and Technical Evolution

Over the past decade, TMVR has emerged as a transformative therapy for patients with FMR at elevated or prohibitive surgical risk, particularly those deemed unsuitable for TEER due to complex anatomies or concerns regarding long-term durability [86,87]. Unlike transcatheter aortic valve replacement (TAVR), TMVR must contend with the highly variable geometry of the mitral annulus, the dynamic sub-valvular apparatus, and the proximity of the LVOT. These anatomical features need sophisticated anchoring strategies, precise deployment control, and tailored device sizing.

**Figure 3:**
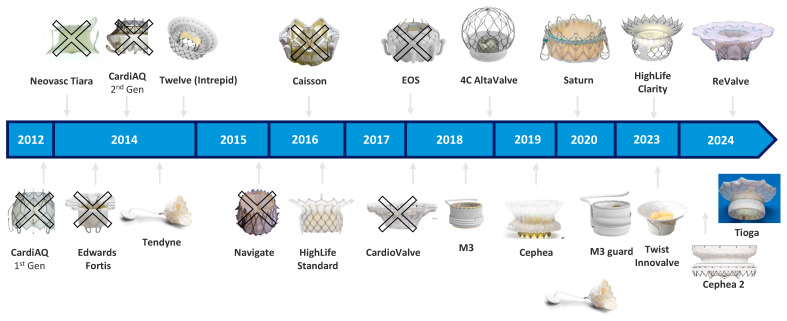
Timeline of transcatheter mitral valve replacement devices (2012–2024). Adapted from TCT 2024 presentations by Granada and M. Leon.

Initial TMVR platforms were conceptualized based on the idea that suitable candidates would primarily exhibit large, non-calcified annuli and dilated ventricles with low LVEF. This design paradigm led to the development of large-profile prostheses with limited radial force and diverse anchoring mechanisms, often requiring transapical delivery. However, real-world registry data—most notably from the Global CHOICE-MI registry—challenged these assumptions, revealing that approximately 70% of screened patients were ineligible due to factors such as small annuli, high LVOT obstruction risk, or unfavorable ventricular anatomy [88,89]. In response, newer-generation TMVR systems shifted toward fully percutaneous, transseptal delivery systems and refined device architecture. These include lower-profile delivery systems, LVOT-sparing designs, and frame geometries adapted to the non-circular and asymmetric nature of the mitral annulus. Innovations in anchoring dynamics and supra-annular positioning now enable successful deployment across a wider range of anatomies, including smaller ventricles and elliptical annuli. A detailed overview of TMVR device development over time is provided in Figure 3.

#### 3.2.2. Overview of Contemporary TMVR Devices

The structural and procedural characteristics of currently available systems are summarized in Table 4.

##### Tiara (Neovasc)

The Tiara TMVR system is characterized by a self-expanding nitinol frame, a trileaflet bovine pericardial valve, a D-shaped frame to match the native mitral annulus, an atrial skirt to minimize paravalvular leak, and three ventricular anchoring tabs for secure fixation. Current clinical evidence for the Tiara system is based on preclinical studies, first-in-human case reports, and early feasibility studies. Initial human implantations demonstrated technical feasibility, procedural safety, and excellent hemodynamic performance, with immediate and sustained elimination of mitral regurgitation, low transvalvular gradients, and no significant LVOT obstruction or paravalvular leak. Early clinical experience has been limited to high-risk or inoperable patients, with successful device implantation and favorable short-term outcomes [89,90,91,92]. Ongoing feasibility studies (TIARA-I and TIARA-II) are evaluating both transapical and future transseptal systems.

##### Tendyne (Abbott Structural)

Tendyne was the first TMVR device to receive CE mark approval (2020) for treatment of native mitral valve disease. It is a self-expanding, tri-leaflet porcine pericardial valve mounted on a nitinol frame, delivered via a transapical approach. The valve is tethered to an epicardial pad at the LV apex, allowing repositioning and retrieval. A single inner valve paired with multiple outer frame sizes allows accommodation of a wide range of annular anatomies. Clinical studies showed 97% technical success, near-complete MR elimination, and reduced HF hospitalizations, although overall mortality remains high [93].

##### Intrepid (Medtronic)

The Intrepid TMVR system consists of a self-expanding tri-leaflet bovine pericardial valve within a dual stent design: a conformable outer ring for annular engagement and a circular inner frame housing the valve. Originally implanted via transapical access, a 29 Fr transfemoral system is now in advanced development. Early studies of the transapical system demonstrated high technical success (96% in the global pilot study), with near-complete elimination of MR and significant improvement in NYHA functional class and quality of life at follow-up. However, early mortality and bleeding rates were notable, largely related to the access route [54]. At 2 years, over half of the patients were alive, with 82% in NYHA class I/II and all available echocardiograms showing ≤mild MR [94]. The transfemoral transseptal system showed 93.9% implantation success in early feasibility studies, with all patients having ≤mild MR and no/trace paravalvular leak at 1 year. The 1-year all-cause mortality was 6.7%, and 91.7% of survivors were in NYHA class I/II, with a median mitral valve mean gradient of 4.6 mm Hg [95,96]. The APOLLO trial is a pivotal, ongoing, randomized study comparing the Intrepid TMVR system to TEER.

##### HighLife (HighLife SAS)

The HighLife TMVR system is characterized by a two-component design: a subannular ring implanted retrogradely via the femoral artery and a transeptally delivered valve that anchors to this ring. The posteriorly directed trajectory preserves the neo-LVOT and minimizes the risk of LVOT obstruction. Clinical data from the HighLife TMVR feasibility study demonstrated technical success in 90% of patients and 30-day device success in 83%. At 1 year, all implanted patients had no or trace (78%) or mild (22%) residual MR, with a mean transmitral gradient of 5.1 mmHg. Notably, no cases of LVOT obstruction were observed, and the mean LVOT gradient remained low at 2.0 mmHg, even in patients with neo-LVOT areas as small as 113 mm^2^ at 1-month post-implant. Additionally, no MV reinterventions were required during follow-up [97,98].

##### Sapien M3 (Edwards Lifesciences)

The Sapien M3 TMVR system combines a balloon-expandable bovine pericardial valve mounted on a cobalt–chromium frame with a nitinol docking system encircling the mitral leaflets. Delivered transeptally, it enables controlled implantation within the docking frame. Early clinical outcomes from feasibility studies and registries show 87% technical success and 2.9% 30-day mortality, with the majority of patients experiencing no or mild residual MR [99].

##### AltaValve (4C Medical Technologies)

The AltaValve TMVR system features a spherical self-expanding nitinol frame designed for supra-annular implantation with atrial anchoring. The valve is suspended above the annulus, avoiding sub-valvular contact and annular oversizing. Both transapical and transseptal delivery systems are under investigation. Its design mitigates LVOT obstruction and maintains annular integrity [100]. Early feasibility studies are ongoing.

##### Evoque Eos (Edwards Lifesciences)

The Evoque Eos valve comprises a trileaflet bovine pericardial valve mounted on a self-expanding nitinol frame. It is delivered via a transseptal approach and adopts an anchoring mechanism that engages the annulus, leaflets, and subvalvular apparatus. A low-profile design with an intra-annular sealing skirt aims to reduce paravalvular leak. Early first-in-human experience in 14 high- or prohibitive-risk patients with moderate or greater MR demonstrated 93% technical success and elimination of mitral regurgitation in 80% of cases at 30 days [101].

##### Innovalve

The Innovalve TMVR system employs a novel LVOT-sparing design characterized by a rotational atrial flange equipped with multiple arms that cinch the chordae tendineae and retract the basal portion of the LV. This mechanism induces a posterior tilt of the prosthesis, thereby facilitating bidirectional mitral flow while avoiding anterior mitral leaflet displacement into the LVOT—a primary cause of obstruction with other TMVR platforms. By preserving anterior flow continuity and maintaining physiological intraventricular flow dynamics, the system promotes efficient LVEF and minimizes blood stasis—a hemodynamic profile that was confirmed in preclinical studies [102].

##### Other Investigational Systems

Additional TMVR platforms under investigation include Cardiovalve (dual nitinol frame), Cephea (double-disc anchoring with a central tri-leaflet valve), NaviGate (self-expanding valve with atrial and ventricular components), and MValve (transseptal docking platform). These devices seek to broaden anatomic applicability through varied anchoring and sealing strategies.

### 3.3. TMVR in Complex Anatomies: Mitral Annular Calcification and Beyond

Among anatomies historically deemed prohibitive for TMVR, severe mitral annular calcification (MAC) remains one of the most technically challenging [103,104,105,106,107]. The dense, frequently circumferential calcium burden limits device expansion and conformability, while increasing the risk of annular rupture, valve embolization, paravalvular leak, and—most critically—LVOT obstruction. These anatomical and procedural aspects are reflected in clinical outcomes: TMVR in the setting of MAC—most commonly performed as valve-in-MAC (ViMAC) using balloon-expandable aortic valves—is associated with lower technical success (~60%) and elevated 30-day and 1-year mortality rates, often exceeding 30% and 50%, respectively. LVOT obstruction, which may occur in up to 40% of cases, is the principal predictor of early mortality [104,105,108,109]. Preprocedural cardiac computed tomography with virtual valve modeling is now essential for estimating the predicted neo-LVOT area and stratifying risk. A threshold of ≤1.7 cm^2^ for the neo-LVOT has demonstrated high predictive accuracy for obstruction.

To address this, several procedural strategies have emerged. The LAMPOON technique (Laceration of the Anterior Mitral Leaflet to Prevent Outflow Obstruction) enables controlled splitting of the anterior mitral leaflet and has shown procedural success > 90% with minimal morbidity [110,111]. Preemptive alcohol septal ablation may further enlarge the neo-LVOT in anatomies with limited septal clearance. In parallel, MAC-adaptive TMVR systems have expanded therapeutic options [112]. Devices such as Tendyne, Tiara, and Cephea anchor within or above the calcified annulus, avoiding reliance on radial force. AltaValve, with its atrial-only fixation and supra-annular design, minimizes subvalvular interaction—particularly advantageous in small ventricles or asymmetric calcification [105,113].

#### Which Patients Are Best Suited for TEER or TMVR?

The integration of TEER and TMVR into the therapeutic landscape of FMR underscores a paradigm shift from a binary algorithm to a personalized, anatomy-driven strategy. TEER remains the preferred intervention in patients with favorable clinical and anatomical characteristics, particularly those aligning with COAPT-like profiles. However, the rapid evolution of TMVR technologies is expanding the scope of treatable anatomies, challenging the notion that replacement should only be considered in TEER-ineligible patients.

In selected anatomies—such as those characterized by severe leaflet tethering, complex annular geometry, or where long-term durability is paramount—TMVR may offer superior hemodynamic outcomes and represents a viable first-line approach. Nonetheless, eligibility for TMVR remains constrained by anatomical criteria, device-specific limitations, and procedural complexity.

For patients with overlapping or borderline features—not clearly qualifying for either strategy—the role of the multidisciplinary Heart Team becomes central. Advanced multimodality imaging, combined with a comprehensive clinical evaluation, enables nuanced decision-making aligned with individual risk profiles and patient goals. As device iterations progress and clinical evidence accumulates, defining “the right device for the right patient” will remain a dynamic, patient-centered process at the core of precision mitral therapy.

## 4. Conclusions

FMR represents a serious clinical challenge that demands a precision-based approach grounded in pathophysiological insight, multimodal imaging, and multidisciplinary expertise. While GDMT remains the cornerstone of care, transcatheter interventions—particularly M-TEER and emerging TMVR platforms—offer transformative potential in selected patients. Evidence underscores that therapeutic success hinges not solely on anatomical feasibility but on rigorous phenotyping, integration of advanced imaging, and alignment with individual clinical trajectories and goals. The evolving paradigm of FMR management must prioritize personalized strategies to optimize outcomes, mitigate procedural futility, and preserve quality of life. As device technologies mature and selection algorithms are refined, a patient-centered, evidence-driven framework will be essential to advance the field and ensure durable benefit across the spectrum of FMR phenotypes.

## Figures and Tables

**Table 1:** Key clinical and anatomical criteria for patient selection.

Domain	Favorable Criteria	Unfavorable Criteria	Implications for Therapy
**Symptoms**	NYHA II–III, persistent despite GDMT	Asymptomatic or end-stage HF	Consider TEER if symptomatic
**MR Severity**	EROA ≥ 30 mm^2^, regurgitant volume ≥ 45 mL	EROA < 20 mm^2^, regurgitant volume < 30 mL	Severe MR more likely to benefit
**LV Function**	LVEF 25–50%, LVEDVi < 96 mL/m^2^	LVEF < 20%, LV severely dilated	Severe dilation may indicate futility
**Anatomy (TEE 3D)**	Coaptation length ≥ 2 mm, depth < 11 mm, acceptable grasping	Severe tethering, calcification, leaflet fissures	Important for TEER feasibility
**Comorbidities**	Stable, limited burden	Frailty, malignancy, end-stage organ dysfunction	May shift focus to conservative approach
**Myocardial Viability**	Viable myocardium (no transmural LGE on CMR)	Extensive fibrosis, transmural infarct	Viable LV predicts better outcomes
**Heart Team Consensus**	Consensus with shared decision-making	No consensus, uncertain patient goals	Multidisciplinary assessment essential

Abbreviations: FMR—Functional Mitral Regurgitation; NYHA—New York Heart Association; GDMT—Guideline-Directed Medical Therapy; HF—Heart Failure; LVEF—Left Ventricular Ejection Fraction; LVEDVi—Left Ventricular End-Diastolic Volume Index; EROA—Effective Regurgitant Orifice Area; MR—Mitral Regurgitation; TEE—Transesophageal Echocardiography; CMR—Cardiac Magnetic Resonance; LGE—Late Gadolinium Enhancement; LV—Left Ventricle; HT—Heart Team; TEER—Transcatheter Edge-to-Edge Repair.

**Table 2:** Key TEE views used during FMR assessment.

TEE View	Angle	Purpose	What to Assess
**Mid-esophageal 4-chamber (ME 4C)**	0–20°	General orientation; initial transseptal puncture guidance	Visualize interatrial septum and needle during transseptal puncture
**Mid-esophageal bicaval view**	90–110°	Primary guidance for transseptal puncture	Ensure posterior and superior puncture site
**Mid-esophageal 2-chamber (ME 2C)**	60–90°	Secondary guidance for puncture	Confirm adequate puncture height (ideal: 4–5 cm from mitral annulus)
**Mid-esophageal mitral commissural view**	50–70°	Commissural visualization (A1–P1, A3–P3)	Identify location of MR jet and guide medial/lateral clip positioning
**Mid-esophageal long axis (ME LAX)**	120–150°	Anterior–posterior alignment of the clip	Leaflet grasping (typically A2–P2), confirm tissue capture and coaptation
**Transgastric short axis/2D-3D**	0–120° (probe advanced into stomach)	Ventricular perspective of the mitral valve	Assess clip arm orientation and residual MR jet direction
**3D en face (atrial surgeon’s view)**	0° with 3D	Global spatial orientation of mitral valve	Visualize mitral scallops (A1–A3, P1–P3), guide medial/lateral positioning
**Color Doppler (all key views)**	Any	MR jet visualization and quantification	Identify origin and severity of MR pre- and post-clip
**PW/CW Doppler at mitral valve level**	0–150°	Hemodynamic assessment post-clip	Measure mean mitral gradient (concern for iatrogenic stenosis if >5 mmH

**Table 3:** Common procedural adverse events in MITRA-FR and COAPT trials.

Complication	MITRA-FR (%)	COAPT (%)
**Overall procedural complication**	14.6%	8.5%
**Pericardial tamponade**	1.4%	0.7%
**Cardiac perforation**	1.4%	0.4%
**Vascular access complications**	2.4%	1.4%
**Stroke/neurological events**	2.8%	1.2%
**Severe mitral stenosis post-implantation**	Not reported	7.6%
**Conversion to surgical mitral repair**	Not reported	1.4%
**Periprocedural mortality**	0.7%	0.4%

**Table 4:** Currently available TMVR devices.

Device Name	Description	Delivery System	Valve Sizes	Access	Available/Ongoing Studies	Approval Status
**Tendyne**	Symmetrical trileaflet porcine bioprosthetic valve with an outer and inner frame. The valve is anchored by a tether secured by an apical pad; repositionable and retrievable.	34 or 36 Fr depending on valve size	The Tendyne valve is in 13 sizes, 8 standard profile (SP) and 5 low profile (LP). The size is calculated according to the anterior-posterior (AP) diameter, the inter-commisural diameter and the perimeter	Transapical	TENDER investigator-initiated, prospective, multicenter trial SUMMIT trial Randomization between Tendyne and Mitraclip	CE Mark approval in January 2020
**Tiara™**	Self-expanding nitinol frame with a trileaflet bovine pericardial valve. D-shaped configuration to conform to the MV annulus	32 Fr for 35 mm valve 36 Fr for 40 mm valve	35 mm and 40 mm	Transapical	TIARA-I feasibility study	N/A
**Intrepid™**	Trileaflet bovine pericardial valve contained in a self-expanding nitinol frame which has a unique dual structure design consisting of a circular inner stent to house the valve and a conformable outer fixation ring to engage the mitral annular anatomy	35 Fr transapical 35 Fr transseptal	Inner bioprosthetic valve is 27 mm in diameter. Outer stent available in two sizes (42 and 48 mm)	Transapical Transseptal	APOLLO trial	N/A
**AltaValve™**	Self-expanding supra-annular nitinol sphere housing a 27 mm bovine pericardial valve	29 Fr	Three annular ring sizes: 40 mm, 46 mm, and 54 mm	Transseptal		N/A
**EVOQUE™**	Self-expanding nitinol frame with bovine pericardial leaflets and a fabric sealing skirt to prevent PVL	28 Fr	44–48 mm	Transseptal		N/A
**HighLife™**	Two components consisting of a subannular ring implant delivered retrogradely via the femoral artery and aortic valve, and the valve component delivered transseptally	30-F (Capsule) mitral valve delivery system with an 18-F shaft.	28 mm valve and ring	Transseptal		N/A
**Sapien M3™**	Two components consisting of a subvalvular “dock”, which encases the balloon expandable Sapien valve (29 mm)	20 Fr	29 mm valve	Transseptal	ENCIRCLE Trial prospective, single arm, multicenter, pivotal, adaptive design study.	CE Mark approval in Europe
**CardioValve™**	Two nitinol self-expanding frames: atrial and ventricular encasing bovine pericardial leaflets	28 Fr	Three sizes available covering commissural diameters from 36 to 55 mm	Transseptal		N/A
**Cephea™**	Self-expanding nitinol double disc system connected via a central column that houses the bovine pericardial trileafet valve; repositionable and recapturable	28 Fr	Three sizes (32, 36, and 40 mm)	Transseptal		N/A
**Innovalve**	Self-expanding frame (26.5 mm inner diameter providing EOA of 2–2.2 cm^2^), which holds 3 leaflets made of bovine pericardial tissue.	39 Fr	28 or 31 mm	Transseptal		N/A
**Revalve** **(Palmetto)**	Palmetto bovine bioprosthetic valve leverages a helical architecture integrating diverse wire thickness Dedicated valve-in-valveplatform (ReValvingSystem)	Not yet disclosed	Not yet disclosed	Transseptal	First-in-human procedure completed in November 2023	N/A
**Saturn**	Low profile TMVR biopros thesis with a central valve which is mechanically connected to an annular ring	39 Fr for transapical 29 Fr for transeptal	28 and 31 mm	Transapical Transseptal		N/A

## Data Availability

Not applicable.

## Abbreviations

The following abbreviations are used in this manuscript:

Abbreviation	Full Term
AF	Atrial Fibrillation
ARNi	Angiotensin Receptor–Neprilysin Inhibitor
CAD	Coronary Artery Disease
CMR	Cardiac Magnetic Resonance
CRT	Cardiac Resynchronization Therapy
CT	Computed Tomography
EROA	Effective Regurgitant Orifice Area
FMR	Functional Mitral Regurgitation
GDMT	Guideline-Directed Medical Therapy
GLS	Global Longitudinal Strain
HF	Heart Failure
KCCQ-OS	Kansas City Cardiomyopathy Questionnaire–Overall Summary
LGE	Late Gadolinium Enhancement
LVEDVi	Left Ventricular End-Diastolic Volume Index
LVEF	Left Ventricular Ejection Fraction
LVOT	Left Ventricular Outflow Tract
MR	Mitral Regurgitation
NYHA	New York Heart Association
RASi	Renin–Angiotensin System Inhibitor
TAVR	Transcatheter Aortic Valve Replacement
TEER	Transcatheter Edge-to-Edge Repair
TEE	Transesophageal Echocardiography
TMVR	Transcatheter Mitral Valve Replacement
